# Excessive sedentary behaviour during hospitalisations among children and adolescents: a prospective observational study

**DOI:** 10.1007/s00431-026-07059-2

**Published:** 2026-05-16

**Authors:** Lærke Winther, Michelle Stahlhut, Derek John Curtis, Mia Eva Hellum, Karen Næs Aaserud, Signe Vandal Pedersen, Jan Christensen, Morten Tange Kristensen, Thomas Hjuler, Thomas Leth Frandsen, Jette Led Sørensen, Christian Have Dall

**Affiliations:** 1 Mary Elizabeth’s Hospital and Juliane Marie Centre, Copenhagen University Hospital – Rigshospitalet, Copenhagen, Denmark; 2 Centre for Clinical Research and Prevention, Copenhagen University Hospital, Bispebjerg and Frederiksberg Hospital, Copenhagen, Denmark; 3 Child Centre Copenhagen, The Child and Youth Administration, City of Copenhagen, Copenhagen, Denmark; 4 Department of Children and Adolescents With Surgical Diseases of the Face, Bones, and Joints, Copenhagen University Hospital – Rigshospitalet, Copenhagen, Denmark; 5 Department of Paediatrics and Adolescent Medicine, Copenhagen University Hospital – Rigshospitalet, Copenhagen, Denmark; 6 Department of Occupational Therapy and Physiotherapy, Copenhagen University Hospital – Rigshospitalet, Copenhagen, Denmark; 7 Department of Public Health, Section of Social Medicine, University of Copenhagen, Copenhagen, Denmark; 8 Department of Physical and Occupational Therapy, Copenhagen University Hospital, Bispebjerg and Frederiksberg Hospital, Copenhagen, Denmark; 9 Department of Clinical Medicine, University of Copenhagen, Copenhagen, Denmark; 10 Department of Otorhinolaryngology and Audiology, Copenhagen University Hospital – Rigshospitalet, Copenhagen, Denmark

**Keywords:** Accelerometery, Physical activity, Prolonged sedentary behaviour, Surgery

## Abstract

The purpose of this study is to objectively quantify physical activity and sedentary behaviour in children and adolescents during hospital admission using device-based monitoring and investigate the association between sedentary behaviour and pain, constipation, surgery status, movement restrictions, 30-day readmission, and length of stay. Children and adolescents aged 2 ≤ 17 years admitted between March and September 2025 to three paediatric departments at a Danish hospital were eligible for this prospective observational study. Children and adolescents scheduled for day surgery and wheelchair users were excluded. All participants wore two triaxial accelerometers throughout their hospital stay. In total, 103 hospitalised children and adolescents were included. The participants spent 45 min per day (interquartile range 20–125) in upright position (standing and walking time combined) and were sedentary (sitting and lying time combined) for 23.3 h per day (21.8–23.7). Adolescents undergoing spine surgery were sedentary for 23.6 h per day (23.5–23.7), of which 22.6 h (21.8–23.0) were spent lying in bed. Sedentary behaviour was significantly associated with higher age (*p* < 0.001), opioid consumption beyond standardised analgesia (p = 0.028), and orthopaedic procedures (*p* < 0.001). No associations between sedentary behaviour and length of stay (*p* = 0.391), 30-day readmission (*p* = 0.467), laxative use (*p* = 0.695), and postoperative movement restrictions (*p* = 0.637) were found.

*Conclusion*: Hospitalised children and adolescents in this study exhibited high levels of sedentary behaviour. The findings highlight that mobilisation strategies are needed across all paediatric surgical procedures, but adolescents, patients undergoing orthopaedic or spine procedures, and those prescribed opioids beyond standardised analgesia require particular attention. There is a need for further evidence of the consequences of sedentary behaviour during hospitalisation among children and adolescents.

**Table:** 

**What is Known:** • *Prolonged sedentary behaviour is associated with adverse health outcomes in healthy children and adolescents and in hospitalised adults.* • *Accelerometery provides objective, validated measurements of physical activity and sedentary behaviour in children and adolescents, with advantages over self-report methods.*
**What is New:** • *These findings highlight low levels of physical activity (767 steps/day) and high levels of sedentary behaviour (23.3 h/day) among hospitalised children and adolescents.* • *These patterns remain consistent across surgical procedures and age categories.*

**Supplementary Information:**

The online version contains supplementary material available at 10.1007/s00431-026-07059-2.

## Introduction

Physical activity is essential for children’s health, growth, and functional development [1], while prolonged sedentary behaviour is associated with adverse physical and psychosocial outcomes [2]. International guidelines recommend children and adolescents engage in at least 60 min of moderate-to-vigorous physical activity daily regardless of health and disability [3], walk 9000–14,000 steps per day [4, 5], and limit the amount of time spent being sedentary [3]. Yet, inactivity remains common even among healthy populations [6].

Hospitalisation further restricts movement: pain, nausea, fatigue, intravenous lines, environmental barriers, time, staffing resources, and the need for assistance with transfers often confine patients to bed, fostering long periods of inactivity [7–9]. In adults, hospital immobility is known to cause rapid declines in muscle mass, strength, and functional independence, leading to longer recovery times and poorer outcomes [10, 11]. However, much less is known about how hospitalisation affects physical behaviour (umbrella term for physical activity and sedentary behaviour [12]) in children and adolescents.

Existing studies examining physical activity in paediatric inpatients are scarce, typically limited to small or highly specific populations [13–15]. Few have provided objective, device-based measurements across broader diagnostic groups or surgical procedures. As a result, there is limited understanding of how age, type of surgery, and clinical outcomes such as pain and constipation are associated with physical behaviour during hospital stays. Understanding children and adolescents’ physical behaviour in hospital is essential for designing targeted interventions and mobilisation strategies.

The aim of the present study was to objectively quantify physical activity and sedentary behaviour among hospitalised children and adolescents in paediatric departments and, secondarily, to investigate associations between pain, constipation, surgery status, movement restrictions, 30-day readmission, length of stay, and sedentary behaviour.

## Materials and methods

### Study design and setting

This was a prospective observational study conducted at a Danish tertiary hospital. Description of the Danish healthcare system can be found in Online Resource 1, “Clinical context”, table A.

A pragmatic approach was applied by including children and adolescents from multiple subspecialties to capture real-world clinical conditions [16]. The study was conducted across three paediatric departments. The departments comprised orthopaedic, spine, bone tumour, ear-nose-throat, orofacial, and eye surgery (Department 1); abdominal and urogenital surgery (Department 2); and cardiology (Department 3). Further descriptions of the departments can be found in Online Resource 1, “Clinical context”, tables b—c. Patients were recruited from Department 1 between March and September 2025 and from Department 2 and 3 from June to September 2025. Hospitalisations across the three departments were generally short, with few exceptions involving children and adolescents with severe pathology or traumatic events. These patients were more likely to decline participation. The first 14 days were used for pilot-testing the setup. After the pilot-test, we (1) changed plasters from the original SENS plasters to medical tape (3 M Micropore surgical tape) due to discomfort, (2) added the exclusion criteria of lack of cooperation due to mental illness, and (3) decided to recruit on the day of surgery (not during their preparation meeting the day prior to surgery). This study adhered to the Declaration of Helsinki principles [17] and to the Strengthening the Reporting of Observational Studies in Epidemiology (STROBE) guidelines [18]. Written informed consent was obtained from parents/guardians for all participants, and assent from children was sought when possible. Ethical approval was not required under Danish regulations (the Scientific Ethics Committees of the Capital Region, Denmark, F-25009906). Data handling and processing complied with Danish data protection regulations with approval from the Danish Data Security Authority (P-2020—121).

### Participants

Eligibility criteria were children and adolescents aged 2 ≤ 17 years admitted to the departments. Children and adolescents who were scheduled for day surgery, lacked cognitive capacity to cooperate, or habitual wheelchair users were not eligible. Not all children and adolescents underwent surgery; some were admitted for observation (e.g. postoperative bleeding), for intravenous antibiotic treatment, or reduction of limb-oedema prior to cast placement. During hospitalisation, children and adolescents were offered daily mobilisation as standard practice. Nurses managed routine mobilisation, such as assisting with sitting, standing, and walking. Physiotherapists provided specialised treatment when prescribed, addressing individual mobility challenges and rehabilitation needs, and were responsible for assessing and instructing patients in the use of walking aids. No additional mobilisation protocols or interventions were implemented during this study.

### Data collection

Data collectors (project nurses from Department 1 and the primary investigator) assessed children and adolescents’ habitual functional mobility using the Cumulated Ambulation Score (CAS) [19], categorising their ambulation level as “independent”, “with assistance”, or “not able to walk”. Those unable to walk (wheelchair users) were excluded. A medical doctor extracted diagnoses, pain medication, and laxative use from electronic medical records. Patients were invited to participate by data collectors either prior to surgery or upon postoperative ward arrival. Those not undergoing surgery were invited as soon as possible after admission. Families received oral and written information about the study, including a video-demonstration of the accelerometers [20]. Data collectors fitted accelerometers and coded them individually to commence data collection upon ward return, excluding time in the Post-Anaesthesia Care Unit. Data collection terminated at discharge, and families were instructed to remove the accelerometers and return them to a designated mailbox in the department before leaving.

### Variables

The primary outcome was physical behaviour comprising upright time and sedentary behaviour. Upright time was defined as a composite variable combining time spent standing and walking (minutes per day), while sedentary behaviour was defined as a composite variable combining time spent lying and sitting (hours per day) [21]. Secondary outcomes included step count per day, hours per day spent sitting, hours per day spent lying, minutes per day spent standing, minutes per day spent walking, surgery status (operated or not), opioid consumption beyond standardised analgesia (per re nata (PRN) opioid consumption), laxative use, movement restrictions, length of stay, and 30-day readmission.

### Physical behaviour

Physical behaviour was assessed using SENS Motion triaxial accelerometers [22]. The accelerometers used time-sampling intervals of 5—s epochs at 12 Hz sampling frequency and were previously validated for children [23]. One accelerometer was placed on the lateral aspect of the femur, and one placed on the sternum. They monitored time spent walking, standing, sitting, lying, and step count. Further specifications of the accelerometers can be found elsewhere [23]. Valid data was defined as a wear time of > 20 h per hospital admission [24]. Participants with less than 20 h of wear time were excluded from analyses. This pragmatic threshold was necessary given the acute care context.

### Clinical outcomes

PRN opioid consumption was extracted from medical records as a proxy measure for pain. Constipation was documented by clinical nursing staff, with laxative use obtained from the electronic medical administration journals serving as a proxy measure for constipation. The 30-day readmission rate and length of stay were extracted from electronic medical records. Movement restrictions, obtained from the physician’s surgery notes, were categorised as limited activity (low intensity activities for following weeks), non-weight bearing (6 weeks), or strict bed rest (24 h, then unrestricted mobility).

### Data management and analysis

Data from electronic medical records were entered into Research Electronic Data Capture (REDCap) [25, 26], while accelerometer data were stored in SENS web [22]. All data were transferred to R (version 4.5.0; R Foundation for Statistical Computing, Vienna, Austria) and merged. Daily averages from the accelerometers were calculated as (∑activity data/wear time in hours) × 24 h. Subgroup analyses examined age groups (2–5, 6–11, and 12–17 years) and type of surgery defined by anatomical region: head (ear-nose-throat, orofacial, and eye surgery), organ (cardiology, abdomen and urology), orthopaedic (extremities), and spine surgeries.

Activity data violated the assumption of normality and remained non-normally distributed following logarithmic transformation. However, with a sample size exceeding 100, parametric assumptions were robust given the Central Limit Theorem [27]; thus, parametric methods were applied.

Descriptive data were presented as medians and interquartile ranges (IQR), and parametric analyses used means and standard deviations. Analyses included logistic regression (dichotomous outcomes), analysis of variance (ANOVA) (continuous outcomes), and multiple linear regression (factors associated with postoperative sedentary behaviour). Interactions between surgery type and other variables were tested using ANOVA *F*-tests and model comparisons (AIC, adjusted *R*^2^). Type of surgery and age interaction was statistically significant (*p* < 0.05, adjusted *R*^2^ = 0.455) and provided the best model fit. The final model included age, sex, type of surgery, their interaction, surgery status, movement restrictions, and length of hospital stay. Due to small subgroup sizes for age-type of surgery strata (range *n* = 1–19), stratified analyses were not performed; instead, main effects were examined separately with multivariable analysis including interaction terms. Missing accelerometer data, for instance, when devices were removed for imaging, were excluded from the dataset.

## Results

**Fig. 1:**
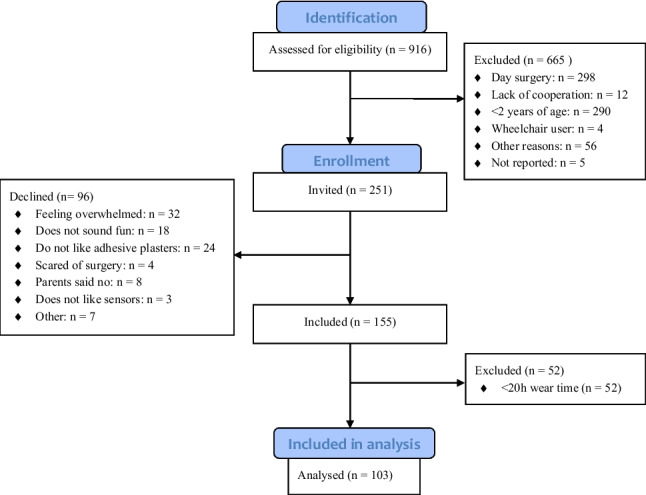
Flow chart of participants in the study. Other reasons for exclusion included not being able to reach expected minimum wear time for a valid day (i.e. acute hospital admissions in the evening and early discharge the following morning), transfer to local hospital or another department, etc

A total of 251 children and adolescents were eligible and invited to participate. Of those, 96 declined the invitation (Fig. 1).

The remaining 155 children and adolescents were fitted with accelerometers. Upon data analysis, 52 were excluded with < 20 h of wear time. Of these, 21 removed the accelerometers early due to itching (*n* = 11) or reasons not reported (*n* = 10). The remaining were discharged before reaching minimum wear time. Thus, 103 children and adolescents were included in the analyses (Table 1). The recruitment acceptance rate was 65% in Dept. 1, 63% in Dept. 2, and 17% in Dept. 3. The cohort comprised children and adolescents with several primary diagnoses, most commonly musculoskeletal (*n* = 46) and ear-nose-throat disorders (*n* = 37), with smaller numbers of cancer, cardiovascular, gastrointestinal, haematological, sensory, urogenital, and other conditions. Many participants had comorbidities, particularly ear-nose-throat (*n* = 38), musculoskeletal (n = 29), and neurological and pulmonary disorders (*n* = 15 each). Overview of diagnoses can be found in Online Resource 2 “Diagnoses”, table a.

**Table 1:** Baseline characteristics. *Head surgery included ear-nose-throat, orofacial, and eye surgery. Organ surgery included abdominal, urological and cardiac surgery. Orthopaedic surgery included orthopaedic and bone tumour surgery. Spine surgery included surgery of the spine. **Based on Cumulated Ambulation Score, Walk category

Baseline characteristics	Overall *N* = 103	Ages 2–5 *n* = 19	Ages 6–11 *n* = 25	Ages 12 ≤ 17 *n* = 59
Age, mean (SD)	11.4 (4.6)	3.5 (1.0)	9.5 (1.3)	14.8 (1.6)
Sex, ***n*** (%)				
Boys	49 (48%)	8 (42%)	16 (64%)	25 (42%)
Girls	54 (52%)	11 (58%)	9 (36%)	34 (58%)
Surgery status, ***n*** (%)				
No surgery	14 (14%)	3 (16%)	2 (8%)	9 (15%)
Type of surgery^*^, ***n*** (%)				
Head surgery	45 (44%)	13 (68%)	13 (50%)	19 (32%)
Organ surgery	10 (10%)	2 (11%)	3 (12%)	5 (8%)
Orthopaedic surgery	30 (29%)	4 (21%)	8 (32%)	18 (31%)
Lower extremities	29 (97%)			
Spine surgery	18 (17%)	0 (0%)	1 (5%)	17 (29%)
Movement restrictions, ***n*** (%)	19 (19%)	4 (21%)	6 (24%)	12 (20%)
Limited activity	4 (4%)	1 (5%)	2 (8%)	1 (2%)
Non-weight bearing	14 (14%)	2 (11%)	3 (12%)	9 (15%)
Strict bed rest	1 (1%)	0 (0%)	0 (0%)	1 (2%)
Preoperative ambulation level, ***n*** (%)^**^				
Independent	99 (93%)	17 (89%)	25 (100%)	57 (97%)
With assistance	4 (3.7%)	2 (11%)	0 (0%)	2 (3%)
Length of stay, days (median (IQR))	3 (1–4)	2 (1–3)	2 (1–3)	3 (1–4.5)
Wear time, hours (median (IQR))	40 (23–54)	25 (21.5–45)	31 (22–47)	47 (24–58)

**Fig. 2:**
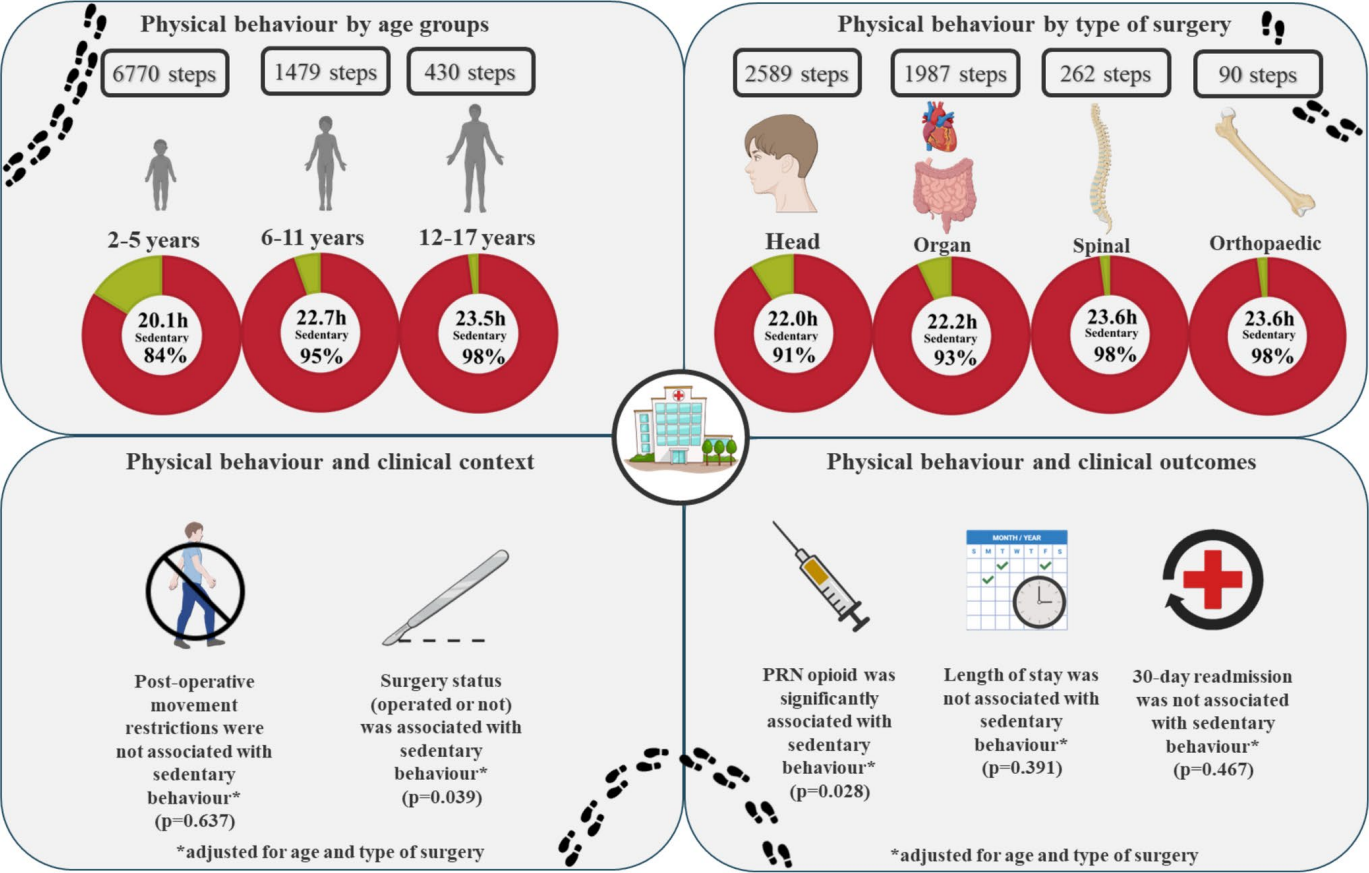
Summary of main results. Steps are reported as medians per day. Sedentary behaviour is reported in median hours and percentage per day. Red, sedentary (sitting/lying). Green, upright (standing/walking). Mean differences with confidence intervals are available in Online Resource 3 “Expanded statistics”, tables a—h

**Fig. 3:**
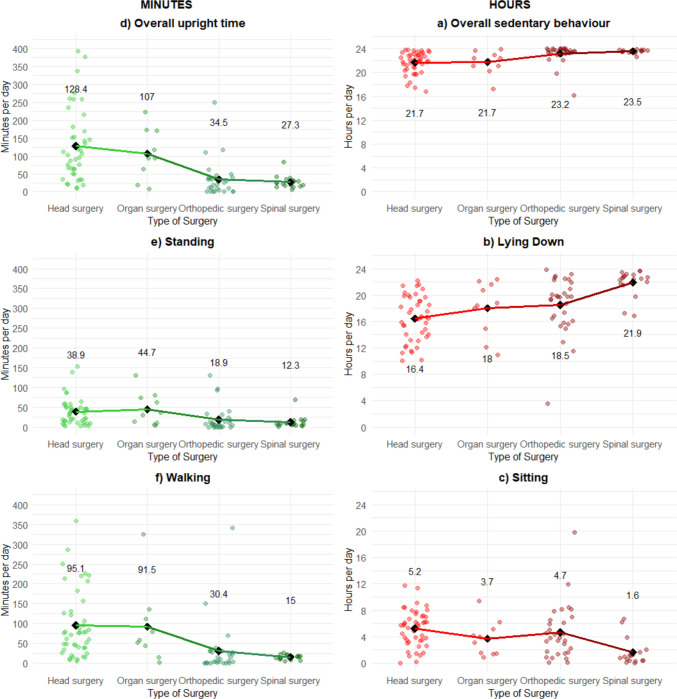
Daily physical behaviour by type of surgery. **a**–**c** Sedentary behaviour (left): **a** overall sedentary time, **b** lying down, and **c** sitting. **d**–**f** Active behaviour (right): **d** overall upright time, **e** standing, and **f** walking. Diamond-shaped markers indicate group means. Mean values are displayed accordingly. Daily physical behaviour by age groups is available in Online Resource 3, “Expanded statistics”, Fig. A

The main results are presented in Fig. 2 and Table 2. In the primary analysis of physical behaviour (Table 2 and Fig. 3), the overall population took a median of 767 steps per day and spent 45 min in an upright position, with 16 min standing and 29 min walking. They were sedentary for a median of 23.3 h, with 18.8 h spent lying in bed and 3.9 h sitting up.

**Table 2:** Analysis of variance (ANOVA) was used to compare steps, upright time, and sedentary behaviour across age groups and surgery types

Comparison of physical behaviour across age groups and types of surgery		Steps Count, median (IQR)	Upright time Minutes, median (IQR)			Sedentary behaviour Hours, median (IQR)		
	***n***	Steps	Overall	Standing	Walking	Overall	Lying	Sitting
All	103	767 (207–2856)	45 (20–125)	16 (7–42)	29 (13–78)	23.3 (21.8–23.7)	18.8 (15.2–21.6)	3.9 (1.5–6.2)
Ages 2–5 (reference)	19	6770 (2708–9296)	236 (142–308)	43 (32–51)	183 (93–225)	20.1 (18.9–21.6)	14.0 (12.1–18.6)	6.1 (2.6–6.9)
Ages 6–11	25	1479 (459–2948)^*^	77 (35–131)^*^	19 (11–58)	46 (30–78)^*^	22.7 (21.8–23.5)^*^	18.5 (14.9–19.9)	3.9 (1.7–7.9)
Ages 12–17	59	430 (107–1664)^*^	32 (16–63)^*^	9 (5–21)^*^	18 (9–34)^*^	23.5 (22.9–23.7)^*^	19.8 (16.9–22.4)^*^	3.2 (1.4–5.7)
Head surgery (reference)	45	2589 (1479–5439)	113 (64–170)	36 (15–50)	76 (38–127)	22.0 (20.8–22.9)	16.5 (14–19)	5.7 (3.4–7.1)
Organ surgery	10	1987 (582–3573)	106 (70–173)	33 (12–71)	69 (45–107)	22.2 (21.1–22.8)	18.6 (15.7–21.4)	3.5 (1.5–4.9)
Orthopaedic surgery	30	90 (4–505)^*^	23 (2–40)^*^	7 (1–16)^*^	13 (1–29)^*^	23.6 (23.3–24)^*^	19.7 (16.1–21.7)^*^	3.8 (1.7–6.4)
Spinal surgery	18	262 (65–413)^*^	24 (17–32)^*^	8 (6–15)^*^	15 (12–18)^*^	23.6 (23.5–23.7)^*^	22.6 (21.8–23.0)^*^	1.1 (0.4–1.6)^*^

* Statistical significance *p* < 0.05

Mean differences and confidence intervals are available in Online Resource 3, “Expanded statistics”, tables a—g.

When stratified by age, the adolescents spent 32 min in upright position and took 430 steps which was significantly less than the youngest children (114 min [63;165], *p* < 0.001, and 6323 steps [4617; 8030], *p* < 0.001, respectively). The youngest children (2–5 years) showed the lowest proportion of sedentary time relative to their peers (Table 2 and Online Resource 3 “Expanded statistics”, Fig. A). Children aged 6–11 and adolescents aged 12–17 years spent significantly more time being sedentary (1.89 h [1.03; 2.77], *p* < 0.001, and 2.76 h [2.01;3.52], *p* < 0.001) compared with the youngest children.

When stratified by type of surgery, children undergoing head surgery were generally the least sedentary group compared to those undergoing organ, orthopaedic, or spine surgery (Table 2). Participants undergoing orthopaedic surgery took 90 steps per day, spent 23 min in upright position, and were lying down for 19.7 h. Those undergoing spine surgery took 262 steps per day, spent 24 min in upright position, were lying down for 22.6 h per day, and sitting up for 1.1 h per day.

**Fig. 4:**
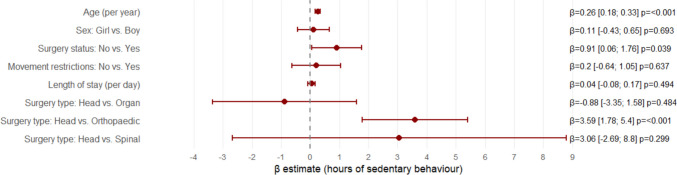
Regression coefficients with 95% confidence intervals for factors associated with postoperative sedentary behaviour (*N* = 103, adjusted *R*^2^ = 0.455). Estimates were adjusted for age, sex, surgery status, movement restrictions, length of hospital stay, and type of surgery*age. Estimates left of zero indicate reduced sedentary behaviour and right of zero indicate increased sedentary behaviour. Increasing age, surgery status, and orthopaedic surgery were significantly associated with increased sedentary behaviour

There was an age effect on type of surgery (interaction plot available in Online Resource 3, “Expanded statistics”, Fig. B), where increased age was associated with increased sedentary behaviour in children and adolescents undergoing head and organ surgeries. The main effects of age and surgery type are reported separately in Table 2 due to small subgroup sizes. Movement restrictions were not associated with sedentary behaviour (ß-coefficient 0.2 [− 0.64; 1.05], *p* = 0.637), which persisted in sensitivity analyses limited to categories with movement restrictions: head and orthopaedic surgeries. The multivariable analysis with interaction terms is presented in Fig. 4.

There was no association between sedentary behaviour and length of stay, either as a predictor (ß-coefficient 0.04 [− 0.08; 0.17], *p* = 0.494) or as an outcome (ß-coefficient 0.13 [− 0.174; 0.442], *p* = 0.391). There was also no association between sedentary behaviour and 30-day readmission (odds ratio (OR) 0.82 [0.47; 1.41], *p* = 0.467) (Online Resource 3, “Expanded statistics”, table h), when adjusting for age and type of surgery, or between sedentary behaviour and laxative use (OR 1.11 [0.69; 2.06], *p* = 0.695), when adjusting for age, type of surgery, and PRN opioid consumption. The association between PRN opioid consumption and sedentary behaviour was statistically significant when adjusting for age and type of surgery (OR 1.75 [1.06; 2.90], *p* = 0.028) (Online Resource 3, “Expanded statistics”, table h).

## Discussion

All children and adolescents exhibited low physical activity and high sedentary behaviour during hospital admission. The cohort took fewer than 800 steps and spent less than one hour in upright position per day. They were sedentary for more than 23 h per day, with almost 19 h spent lying in bed. Older age was associated with greater sedentary behaviour among children and adolescents undergoing head and organ surgery, whereas all patients undergoing orthopaedic and spine surgeries demonstrated consistently high levels of sedentary behaviour. There were no differences between groups with and without movement restrictions, suggesting the clinical context adversely affects sedentary behaviour. Sedentary behaviour showed no associations with length of stay or 30-day readmission when adjusting for age and type of surgery, though PRN opioid consumption was associated with increased sedentary behaviour.

Children and adolescents took substantially fewer steps than recommended and even remained below the 6000-step recommendation for those living with chronic illness or disability [4]. The high levels of sedentary behaviour in this study were similar to hospitalised children and adolescents with oncologic diseases [15]. Adolescents, orthopaedic, and spine patients were more sedentary compared to adult medical [28], pulmonary [19], and hip fracture surgery patients [29]. In healthy children and adolescents, sedentary behaviour increases from age 2 to 12 and stabilises during adolescence [6], consistent with the present study’s findings of increased sedentary behaviour with older age. However, the sedentary behaviour levels in this study far exceed typical patterns for all age groups [6].

Multiple factors appear to limit physical activity during hospitalisation. Physical symptoms, such as pain, nausea, and fatigue [30, 31], may be compounded by environmental barriers, such as lack of chairs [8], clutter and medical equipment in the hallways, lack of space to mobilise [30, 31], and lack of patient motivation, staff resources, fear of movement, and health literacy [30, 32]. While the consequences of hospital immobility are well described for adults [10], evidence in children and adolescents remains limited. In critically ill children in a paediatric intensive care unit, quadriceps thickness decreased by 1.5% per day, and 53% experienced > 10% muscle atrophy of the quadriceps muscle within one week [33]. Similarly, in patients aged 9–13 years with perforated appendicitis, the psoas muscle area decreased by almost 1% per day [34]. These findings suggest that muscle wasting may occur at similar rates in children as in adults [35]. Reduced muscle mass, a key marker of muscle wasting, is associated with decreased physical function and poorer quality of life [36, 37]. In children, this translates to limited ability to engage in play and daily physical activities, leading to social isolation, low self-esteem, and poorer mental, metabolic, and cardiovascular health, and social well-being [38–40]. However, whether muscle wasting occurs in children and adolescents following orthopaedic and spine surgery remains unknown.

Immobility after surgeries may serve as a starting point for long-term lifestyle modifications. In adolescents with surgically treated lower limb fractures, moderate physical activity remained significantly reduced 18 months postoperatively [41]. In the present study, spine surgery patients were lying in bed for 22.6 h per day, representing a group at particularly high risk of reduced mobilisation. Furthermore, in adolescents undergoing spine surgeries, reduced physical activity has been associated with chronic post-surgical pain at 4 months [42], significantly impacting their quality of life [43]. Although adult evidence suggests that physical activity interventions during hospitalisation may reduce functional decline [44], comparable interventional evidence on postoperative pain outcomes in paediatric populations is currently lacking. In this study, PRN opioid consumption was significantly associated with increased sedentary behaviour, consistent with other research linking sedentary behaviour and pain [45, 46]. In adults undergoing spine surgeries, preoperative fear of movement has also been associated with postoperative sedentary behaviour [47]. The clinical impact may extend beyond acute hospitalisation. Time to return to school varies between 2 and 16 weeks for children and adolescents with orthopaedic and spine surgeries [48, 49], resulting in parental work absence for 15–17 days for spine surgery patients [50].

## Strengths and limitations

A pragmatic approach was adopted to data collection, reflecting real-world clinical conditions. Including children from diverse diagnostic groups introduced heterogeneity but enhanced generalisability and clinical relevance, emphasising that mobilisation is a universal need across paediatric populations.

PRN opioid consumption and laxative use served as proxy measures for pain and constipation, respectively. Pain intensity was registered by nurses, but they used several different pain intensity scales (sometimes three different scales on the same patient on the same day), making cross-patient comparisons impossible. Future studies should include a more standardised way of measuring pain intensity. Similarly, constipation was registered inconsistently; laxative use was therefore used as a proxy. However, this measure had limitations as laxatives were given prophylactically within some surgical fields, e.g. spine surgery, and nurses had mandate to dispense it upon clinical indication without physician prescription or registration in the medical administration journal. This underreporting would not capture all laxative use, potentially underestimating the association between sedentary behaviour and constipation. For postoperative movement restrictions, a potential ceiling effect may explain the lack of group differences. Both restricted and unrestricted groups were so inactive during hospitalisation that time spent sedentary clustered near maximum levels (24 h), potentially limiting the capacity to detect between-group differences. When outcome measures cluster at extreme values, true differences become difficult to distinguish, meaning the lack of observed differences may reflect measurement constraints rather than genuine equivalence between groups [51]. It is also acknowledged that the interaction analyses were hypothesis-generating rather than confirmatory given the small sample sizes in the stratified subgroups.

Our findings may underestimate sedentary behaviour as the patients with the most serious conditions, including patients undergoing trauma or cardiac surgeries, declined participation. Conversely, sedentary behaviour among the youngest children may be overestimated, as many were mobilised in wheelchairs rather than walking with crutches (which requires motor control beyond their developmental age). Wheelchair propulsion generates physical activity that was not captured, as the study lacked upper extremity accelerometers due to absent algorithms for interpreting upper extremity movements [23]. Reports of valid accelerometer wear time criteria vary widely (4–16 h on 1–6 days) [24, 52, 53], and moderate reliability typically requires 6–12 days of wear time [52, 54]. However, 75% of patients in this study were admitted for fewer than 4 days, making prolonged wear time infeasible. A pragmatic compromise was adopted with a minimum of 20 consecutive hours per admission being recorded, reflecting the constraints of acute paediatric hospitalisation. Despite these limitations, this study represents the most rigorous assessment feasible in this clinical setting.

## Implication for clinical practice

What can realistically be expected in terms of physical activity during hospitalisation remains uncertain. This study revealed low levels of physical activity and high levels of sedentary behaviour across ages and types of surgeries. These findings indicate that mobilisation should be prioritised universally, suggesting substantial opportunity for system-level improvement. An age effect emerged within head and organ surgeries, suggesting that targeted mobilisation strategies would benefit adolescents in these settings. Orthopaedic and spine surgery patients exhibited the highest levels of sedentary behaviour, warranting integrated activity protocols within care plans. Additionally, the association between PRN opioid consumption and sedentary behaviour suggests that mobilisation strategies should be considered when prescribing PRN opioids. There is a pressing need for evidence examining how sedentary behaviour during hospitalisation impacts paediatric outcomes, particularly regarding postoperative pain and return to school. 

Drawing on clinical experience and discussions with ward staff, a possible factor in diminishing sedentary behaviour could be involvement of caregivers and patient education in pain mechanisms and daily mobilisation. Reconfiguring ward layouts and common areas to reduce bed-centred routines and create clear opportunities for movement could support higher activity levels among hospitalised children. Individualised education and alignment of responsibilities between healthcare professionals should be prioritised [55]. Early mobilisation after paediatric surgery may yield multiple benefits including shorter time to passage of first flatus and faeces [56], shorter lengths of stay, and decreased opioid use [57].

## Conclusion

Hospitalised children and adolescents in this study exhibited low levels of physical activity and high levels of sedentary behaviour (23.3 h per day). The findings highlight that mobilisation strategies are needed across all paediatric surgical procedures, but adolescents, patients undergoing orthopaedic or spine surgeries, and those prescribed PRN opioids require particular attention. Additional evidence of the consequences of sedentary behaviour during hospitalisation among children and adolescents is needed.

## Supplementary Information

Below is the link to the electronic supplementary material.

ESM 1 (DOCX 3.46 MB)

ESM 2 (DOCX 108 KB)

ESM 3 (DOCX 3.58 MB)

## Data Availability

Datasets generated in the present study are not publicly accessible due to Danish data sharing agreements but may be obtained from the corresponding author upon reasonable request.
