# Supplementary material for: Excessive sedentary behaviour during hospitalisations among children and adolescents: a prospective observational study
Source: Eur J Pediatr. 2026 May 16;185(6):402. doi: 10.1007/s00431-026-07059-2 (PMC13179916; doi:10.1007/s00431-026-07059-2)
Supplement: Supplementary file 1 — (DOCX 3.46 MB) [file 431_2026_7059_MOESM1_ESM.docx]

**Online Resource 1, Clinical context**

**Excessive Sedentary Behaviour During Hospitalisations among Children and Adolescents: A Prospective Observational Study**

Lærke Winther^1^, Michelle Stahlhut^2^_,_ Derek John Curtis^3^, Mia Eva Hellum^4^, Karen Næs Aaserud^4^, Signe Vandal Pedersen^5^, Jan Christensen^6^, Morten Tange Kristensen^7,8^, Thomas Hjuler^4^, Thomas Leth Frandsen^1^, Jette Led Sørensen^1,8^, Christian Have Dall^7,8^

^1^ Mary Elizabeth’s Hospital and Juliane Marie Centre, Copenhagen University Hospital – Rigshospitalet, Copenhagen, Denmark
^2^ Centre for Clinical Research and Prevention, Copenhagen University Hospital, Bispebjerg and Frederiksberg Hospital, Copenhagen, Denmark

^3^ Child Centre Copenhagen, The Child and Youth Administration, City of Copenhagen, Copenhagen, Denmark

^4^Department of Paediatric Surgery, Copenhagen University Hospital – Rigshospitalet, Denmark

^5^Department of Children and Adolescents, Copenhagen University Hospital – Rigshospitalet, Denmark

^6^Department of Occupational Therapy and Physiotherapy, Copenhagen University Hospital –Rigshospitalet

^7^ Department of Occupational Therapy and Physiotherapy, Copenhagen University Hospital, Bispebjerg and Frederiksberg Hospital, Copenhagen, Denmark

^8^ Department of Clinical Medicine, University of Copenhagen, Copenhagen, Denmark

Corresponding author: Laerke Winther, laerke.winther@regionh.dk

The clinical context is described based on the Consolidated Framework for Implementation Science (CFIR)^^[[1]](#footnote-1)^^.

| **Outer setting** | |
| --- | --- |
| **General description of the Danish healthcare system** | The Danish healthcare system is publicly financed, meaning that most healthcare services are funded through taxes. Citizens in Denmark have free access to general practitioners, specialists, emergency departments, hospitals, and acute care.  Denmark is divided into five (four from 2027) regions, which are responsible for running hospitals, psychiatric care, and primary care (general practitioners, specialists, etc.). The municipalities are responsible for prevention, health services for children, rehabilitation, nursing homes, and home care.  The healthcare system operates according to a *gatekeeper model*, where the general practitioner is the citizen’s first point of contact. The general practitioners have the authority to refer patients to other healthcare services such as specialists or physiotherapy. The referral ensures appropriate care pathways, efficient use of public resources, and equal access to healthcare. |
| **Organisation** | **Rigshospitalet** is Denmark’s largest highly specialised university hospital, with nationwide responsibilities (including Greenland and the Faroe Islands) in the management of complex or rare diseases.  Rigshospitalet is organised into a centre structure, where related specialties and treatment functions are gathered under a unified leadership. Each center is headed by a deputy director with overall financial and managerial responsibility. Under the deputy director, clinical departments are led by clinical directors and head nurses. The aim of the center structure is to strengthen interdisciplinary collaboration, efficient management, research, and specialization.  **Juliane Marie Center (JMC)**  This is the hospital’s center for women, children, adolescents, and reproductive health. JMC consists of eight inpatient units with associated outpatient clinics:   - Gynaecology - Obstetrics - Fertility - Neonatology - Paediatrics & Adolescents - Surgical Paediatrics - Growth & Reproduction - Anaesthesia & Surgery   Additionally, JMC hosts several cross-disciplinary units and knowledge centers, including adolescent medicine, the Paediatric Pain Unit, clinical dieticians, physiotherapists and occupational therapists, paediatric palliative care, research units, psychologists, social workers, and pedagogues. |
| **External Supportive Services** | Rigshospitalet collaborates with several volunteer and social organizations that aim to improve everyday life for hospitalized children, adolescents, and families.   - **Legeheltene (Play Heroes)**: Focus on play and physical activity to strengthen well-being, normalcy, and quality of life. - **Danske Hospitalsklovne (Danish Hospital Clowns)**: Provide presence, humour, and imaginative interaction to create joyful breaks from illness and treatment. - **Børnecancerfonden**: Funds coordinator positions supporting families affected by childhood cancer. - **Hr. Berg**: A youth-support programme for 12–24-year-olds with serious, acute, or chronic illnesses, offering social spaces, activities, and peer connection. - **Smilet**: A creative workshop where patients can relax, create, and receive support from pedagogues—also available for patients staying in their rooms. - **MusikBeriget & SpilleBillen**: Live music at the ward or bedside, offering calm, relief, and distraction. - **Oasen**: Rhythmic activities   These initiatives collectively help create a more holistic and humane hospital experience, supporting psychological well-being, social interaction, normalcy, and family needs. |
| **Referral and Admission Pathways** | Patients admitted to Rigshospitalet come either through:   - **Elective pathways** – planned admissions based on referrals from general practitioners, specialists, or other hospitals seeking highly specialised treatment. - **Acute pathways** – urgent admissions without prior referral.   Acute admissions begin with immediate treatment, whereas elective admissions involve planning, preparation, and often outpatient assessment. Both pathways end with a follow-up plan, either at the outpatient clinic, with the general practitioners, or through municipal services (home nursing, rehabilitation). |
| **External Policy & Incentives** | Rigshospitalet operates under a comprehensive set of external policies and financial incentives from both national and regional levels. National quality targets, established through negotiated agreements between the state, the Danish Regions, and the Capital Region, are implemented throughout the hospital and monitored and published annually. The hospital's performance on these targets affects regional funding through the national funding mechanism, which incentivizes the transition from hospital-based to primary and home-based care, as well as digitally supported and integrated care. Furthermore, the hospital operates under national clinical guidelines and standard treatment pathways established by the Danish Health Authority, which the hospital must follow, and whose compliance is monitored. Finally, the hospital faces competition through patients' right to choose among hospitals and the possibility of seeking treatment abroad, which drives incentives to maintain high quality standards on published indicators. |
| **References** | <https://www.commonwealthfund.org/international-health-policy-center/countries/denmark> |

Table a. Description of the outer setting of the hospital based on CFIR.

| **Inner Setting** | | | |
| --- | --- | --- | --- |
| **Department no.** | **Surgical specialities** | **Beds/rooms** | **Activities** |
| **1, surgical diseases of the face, bones and joints** | Plastic surgery  Orthopaedic surgery  Ear-Nose-Throat surgery  Maxillofacial surgery  Ophthalmic surgery | 20 beds, 20 rooms.  All patients are offered single rooms. Each room has two beds (one for the patient, one for the admitted parent), a small table, and two shell chairs. Ten armchairs are available for patients who cannot sit in standard chairs. | A playroom with cushions, swing, slide, cars, and toys.  Multiple bicycles and ride-on vehicles. The floors have road-themed vinyl, and wall graphics create “stations” like post office or police station.  A table tennis room.  A creative room, offering drawing, painting, cutting/gluing, and other crafts. |
| **2, surgical diseases of the abdomen and urogenital area** | Abdominal surgery  Urogenital surgery | 13 beds, 5 rooms.  The rooms are often shared between 2-3 patients. Few single bed rooms are available. | A playroom with a creative station and toys  A pedagogue who facilitates play |
| **3, paediatric heart diseases** | Cardiac diseases and surgery | 9 beds, 5 rooms. The patients will most often share room with another family. | A playroom with toys  Bicycles and ride-on vehicles |

Table b. Structural characteristics of the inner setting of the hospital departments based on CFIR.

| **Inner setting** | |
| --- | --- |
| **Activity facilities** | Activities are used as part of treatment to assess physical ability before and after surgery and to reduce waiting-time frustration.  Meals are served in the department’s kitchen areas, where common dining areas are available. The meals are often collected from the kitchen areas and brought to the rooms. |
| **Networks & Communications** | **Preoperative preparation for elective surgery includes:**   - Consultation with the surgeon - Consultation with a ward nurse - Consultation with an anaesthesiologist - *Optional*: consultation with a physiotherapist (mandatory for major spine or orthopaedic surgery)   The physiotherapist introduces exercises that can be practiced before surgery. The nurse’s task is to align expectations with the patient and family, explain what to expect, and help reduce anxiety and insecurity. Written information and a welcome brochure are provided. |
| **Culture** | Mobilisation in the department is structured around the individual patient’s care pathway. Nurses oversee routine daily mobilisation such as transfers from bed to chair and small walks.  For pathways requiring specialised postoperative mobilisation:   - A referral is made to the physiotherapists by the surgeon or nurse. - The physiotherapist evaluates frequency, activities, and required assistive devices. - The physiotherapist and nurse coordinate mobilisation timing. - Nurses ensure optimal pain management beforehand. - Mobilisation is carried out in collaboration with the family. - In the evenings, mobilisation is handled by the nurse and family.   Rehabilitation plans upon discharge are prepared jointly by the surgeon and physiotherapist. Assistive devices or adjustments to patients’ own equipment are the responsibility of the physiotherapist. |

Table c. Description of inner setting of the hospital departments based on CFIR.

1. Damschroder, L.J., Aron, D.C., Keith, R.E. *et al.* Fostering implementation of health services research findings into practice: a consolidated framework for advancing implementation science. *Implementation Sci* **4**, 50 (2009). <https://doi.org/10.1186/1748-5908-4-50> [↑](#footnote-ref-1)
