# Supplementary material for: Excessive sedentary behaviour during hospitalisations among children and adolescents: a prospective observational study
Source: Eur J Pediatr. 2026 May 16;185(6):402. doi: 10.1007/s00431-026-07059-2 (PMC13179916; doi:10.1007/s00431-026-07059-2)
Supplement: Supplementary file 2 — (DOCX 108 KB) [file 431_2026_7059_MOESM2_ESM.docx]

**Online Ressource 2, Diagnoses**

**Excessive Sedentary Behaviour During Hospitalisations among Children and Adolescents: A Prospective Observational Study**

Lærke Winther^1^, Michelle Stahlhut^2^_,_ Derek John Curtis^3^, Mia Eva Hellum^4^, Karen Næs Aaserud^4^, Signe Vandal Pedersen^5^, Jan Christensen^6^, Morten Tange Kristensen^7,8^, Thomas Hjuler^4^, Thomas Leth Frandsen^1^, Jette Led Sørensen^1,8^, Christian Have Dall^7,8^

^1^ Mary Elizabeth’s Hospital and Juliane Marie Centre, Copenhagen University Hospital – Rigshospitalet, Copenhagen, Denmark
^2^ Centre for Clinical Research and Prevention, Copenhagen University Hospital, Bispebjerg and Frederiksberg Hospital, Copenhagen, Denmark

^3^ Child Centre Copenhagen, The Child and Youth Administration, City of Copenhagen, Copenhagen, Denmark

^4^Department of Paediatric Surgery, Copenhagen University Hospital – Rigshospitalet, Denmark

^5^Department of Children and Adolescents, Copenhagen University Hospital – Rigshospitalet, Denmark

^6^Department of Occupational Therapy and Physiotherapy, Copenhagen University Hospital –Rigshospitalet

^7^ Department of Occupational Therapy and Physiotherapy, Copenhagen University Hospital, Bispebjerg and Frederiksberg Hospital, Copenhagen, Denmark

^8^ Department of Clinical Medicine, University of Copenhagen, Copenhagen, Denmark

Corresponding author: Laerke Winther, [laerke.winther@regionh.dk](mailto:laerke.winther@regionh.dk)

| **Overview of diagnostic categories** | |
| --- | --- |
| **Acute conditions** | n |
| Acute primary diagnoses | 33/103 |
|  |  |
| **Primary diagnoses** | n |
| Cancer | 3 |
| Cardiovascular disorder | 2 |
| Ear-Nose-Throat disorders | 37 |
| Gastrointestinal disorders | 5 |
| Haematological disorders | 2 |
| Muscoloskeletal disorders | 46 |
| Sensory impairment | 1 |
| Urogenital disorders | 3 |
| Other | 4 |
|  |  |
| **Comorbidities** |  |
| Cardiovascular disorders | 9 |
| Ear-Nose-Throat disorders | 38 |
| Endocrinological disorders | 8 |
| Gastrointestinal disorders | 11 |
| Haematological disorders | 2 |
| Muscoloskeletal disorders | 29 |
| Neurological disorders | 15 |
| Cerebral palsy | 2 |
| Pulmonary disorders | 15 |
| Sensory impairment | 15 |
| Syndromes/congenital malformation | 8 |
| Urogenital disorders | 10 |
| Other | 36 |
| Table a. Categories are based on SKS-codes (Danish National Patient Registry classification system). | |
