# Supplementary material for: Excessive sedentary behaviour during hospitalisations among children and adolescents: a prospective observational study
Source: Eur J Pediatr. 2026 May 16;185(6):402. doi: 10.1007/s00431-026-07059-2 (PMC13179916; doi:10.1007/s00431-026-07059-2)
Supplement: Supplementary file 3 — (DOCX 3.58 MB) [file 431_2026_7059_MOESM3_ESM.docx]

**Online Resource 3, Expanded statistics**

**Excessive Sedentary Behaviour During Hospitalisations among Children and Adolescents: A Prospective Observational Study**

Lærke Winther^1^, Michelle Stahlhut^2^_,_ Derek John Curtis^3^, Mia Eva Hellum^4^, Karen Næs Aaserud^4^, Signe Vandal Pedersen^5^, Jan Christensen^6^, Morten Tange Kristensen^7,8^, Thomas Hjuler^4^, Thomas Leth Frandsen^1^, Jette Led Sørensen^1,8^, Christian Have Dall^7,8^

^1^ Mary Elizabeth’s Hospital and Juliane Marie Centre, Copenhagen University Hospital – Rigshospitalet, Copenhagen, Denmark
^2^ Centre for Clinical Research and Prevention, Copenhagen University Hospital, Bispebjerg and Frederiksberg Hospital, Copenhagen, Denmark

^3^ Child Centre Copenhagen, The Child and Youth Administration, City of Copenhagen, Copenhagen, Denmark

^4^Department of Paediatric Surgery, Copenhagen University Hospital – Rigshospitalet, Denmark

^5^Department of Children and Adolescents, Copenhagen University Hospital – Rigshospitalet, Denmark

^6^Department of Occupational Therapy and Physiotherapy, Copenhagen University Hospital –Rigshospitalet

^7^ Department of Occupational Therapy and Physiotherapy, Copenhagen University Hospital, Bispebjerg and Frederiksberg Hospital, Copenhagen, Denmark

^8^ Department of Clinical Medicine, University of Copenhagen, Copenhagen, Denmark

Corresponding author: Laerke Winther, laerke.winther@regionh.dk

| **Sedentary behaviour** | | | | |
| --- | --- | --- | --- | --- |
|  | **n** | **Overall,**  **hours, mean (SD)** | **Estimated mean difference [CI95]** | **p-value** |
| **Ages 2-5** (reference) | 19 | 20.4 (2.3) |  |  |
| **Ages 6-11** | 25 | 22.30 (1.65) | 1.89 [1.03;2.77] | <0.001 |
| **Ages 12-17** | 59 | 23.17 (0.87) | 2.76 [2.01;3.52] | <0.001 |
| **Head surgery** (reference) | 45 | 21.67 (1.79) |  |  |
| **Organ surgery** | 10 | 21.72 (1.94) | 0.05 [-1.05;1.14] | 0.935 |
| **Orthopaedic surgery** | 30 | 23.19 (1.57) | 1.51 [0.78;2.25] | <0.001 |
| **Spine surgery** | 18 | 23.55 (0.28) | 1.87 [0.99;2.747] | <0.001 |

**Table a:** Results of ANOVA for overall sedentary behavior in hours per day (24h). Significance levels (p-values) are reported in table 2 in the main article.

| **Sedentary behaviour** | | | | |
| --- | --- | --- | --- | --- |
|  | **n** | **Lying,**  **mean (SD)** | **Estimated mean difference [CI95]** | **p-value** |
| **Ages 2-5** (reference) | 19 | 15.4 (4.3) |  |  |
| **Ages 6-11** | 25 | 17.2 (3.7) | 1.8 [-0.4;4.1] | 0.106 |
| **Ages 12-17** | 59 | 19.4 (3.5) | 4.0 [2.1;6.0] | <0.001 |
| **Head surgery** (reference) | 45 | 16.4 (3.4) |  |  |
| **Organ surgery** | 10 | 18.0 (4.1) | 1.6 [-0.9;4.0] | 0.208 |
| **Orthopaedic surgery** | 30 | 18.5 (4.2) | 2.1 [0.4;3.7] | 0.014 |
| **Spine surgery** | 18 | 21.9 (2.0) | 5.5 [3.5;7.4] | <0.001 |
|  | | | | |

**Table b:** Results of ANOVA for lying sedentary behavior in hours per day (24h). Significance levels (p-values) are reported in table 2 in the main article.

| **Sedentary behaviour** | | | | |
| --- | --- | --- | --- | --- |
|  | **n** | **Sitting,**  **mean (SD)** | **Estimated mean difference [CI95]** | **p-value** |
| **Ages 2-5** (reference) | 19 | 5.0 (3.0) |  |  |
| **Ages 6-11** | 25 | 5.1 (3.6) | 0.1 [-1.9;2.1] | 0.950 |
| **Ages 12-17** | 59 | 3.7 (3.3) | -1.3 [-3.0;0.5] | 0.148 |
| **Head surgery** (reference) | 45 | 5.2 (2.8) |  |  |
| **Organ surgery** | 10 | 3.7 (2.7) | -1.5 [-3.7;0.6] | 0.165 |
| **Orthopaedic surgery** | 30 | 4.7 (4.1) | -0.6 [-2.0;0.9] | 0.438 |
| **Spine surgery** | 18 | 1.6 (2.0) | -3.6 [-5.3;1.9] | <0.001 |
|  | | | | |

**Table c:** Results of ANOVA for sitting sedentary behavior in hours per day (24h). Significance levels (p-values) are reported in table 2 in the main article.

| **Upright time** | | | | |
| --- | --- | --- | --- | --- |
|  | **n** | **Overall, minutes,**  **mean (SD)** | **Estimated mean difference [CI95]** | **p-value** |
| **Ages 2-5** (reference) | 19 | 216 (140) |  |  |
| **Ages 6-11** | 25 | 102 (99) | -114 [-165;-63] | <0.001 |
| **Ages 12-17** | 59 | 47 (45) | -169 [-213;-125] | <0.001 |
| **Head surgery** (reference) | 45 | 135 (107) |  |  |
| **Organ surgery** | 10 | 137 (116) | 2 [-64;67] | 0.956 |
| **Orthopaedic surgery** | 30 | 49 (94) | -86 [-130;-42] | <0.001 |
| **Spine surgery** | 18 | 27 (17) | -108 [-160;-56] | <0.001 |

**Table d:** Results of ANOVA for overall upright time in minutes per day (24h). Significance levels (p-values) are reported in table 2 in the main article.

| **Upright time** | | | | |
| --- | --- | --- | --- | --- |
|  | **n** | **Walking, mean (SD)** | **Estimated mean difference [CI95]** | **p-value** |
| **Ages 2-5** (reference) | 19 | 163 (111) |  |  |
| **Ages 6-11** | 25 | 64 (67) | -99 [-136;-62] | <0.001 |
| **Ages 12-17** | 59 | 28 (30) | -135 [-167;-103] | <0.001 |
| **Head surgery** (reference) | 45 | 95 (85) |  |  |
| **Organ surgery** | 10 | 91 (92) | -4 [-54;47] | 0.886 |
| **Orthopaedic surgery** | 30 | 30 (66) | -65 [-99;-31] | <0.001 |
| **Spine surgery** | 18 | 15 (7) | -80 [-120;-40] | <0.001 |
| **Table e:** Results of ANOVA for walking upright time in minutes per day (24h). Significance levels (p-values) are reported in table 2 in the main article. | | | | |
| **Upright time** | | | | |
|  | **n** | **Standing, mean (SD)** | **Estimated mean difference [CI95]** | **p-value** |
| **Ages 2-5** (reference) | 19 | 50 (41) |  |  |
| **Ages 6-11** | 25 | 38 (40) | -12 [-30;7) | 0.216 |
| **Ages 12-17** | 59 | 18 (22) | -31 [-47;-15] | <0.001 |
| **Head surgery** (reference) | 45 | 39 (33) |  |  |
| **Organ surgery** | 10 | 45 (41) | 6 [-16;28] | 0.595 |
| **Orthopaedic surgery** | 30 | 19 (32) | -20 [-35;-5] | 0.007 |
| **Spine surgery** | 18 | 12 (15) | -27 [-44;-9] | 0.002 |
|  | | | | |

**Table f:** Results of ANOVA for standing upright time in minutes per day (24h). Significance levels (p-values) are reported in table 2 in the main article.

|  | **n** | **Steps, mean (SD)** | **Estimated mean difference [CI95]** | **p-value** |
| --- | --- | --- | --- | --- |
| **Ages 2-5** (reference) | 19 | 7387 (6638) |  |  |
| **Ages 6-11** | 25 | 2160 (2505) | -5227 [-7197;-3258] | <0.001 |
| **Ages 12-17** | 59 | 1064 (1437) | -6323 [-8030;-4617] | <0.001 |
| **Head surgery** (reference) | 45 | 4119 (4679) |  |  |
| **Organ surgery** | 10 | 3619 (5115) | -498 [-3070;2071] | 0.700 |
| **Orthopaedic surgery** | 30 | 947 (2322) | -3172 [-4905;-1439] | <0.001 |
| **Spine surgery** | 18 | 397 (512) | -3722 [-5773;-1672] | <0.001 |
|  | | | | |

**Table g:** Results of ANOVA for steps per day (24h). Significance levels (p-values) are reported in table 2 in the main article.


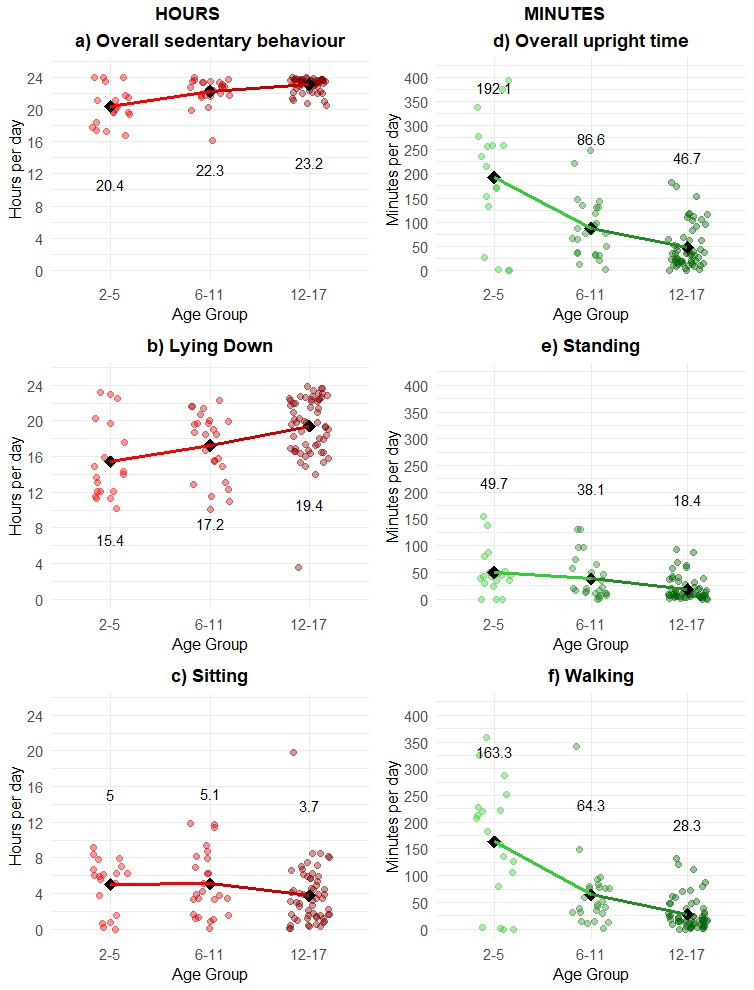


**Fig. A**. Daily physical behaviour by age group. Figures a–c show sedentary behaviour (left): (a) overall sedentary time, (b) lying down, and (c) sitting. Figures d–f show active behaviour (right): (d) overall upright time, (e) standing, and (f) walking. Diamond-shaped markers indicate group means. Mean values are displayed accordingly.


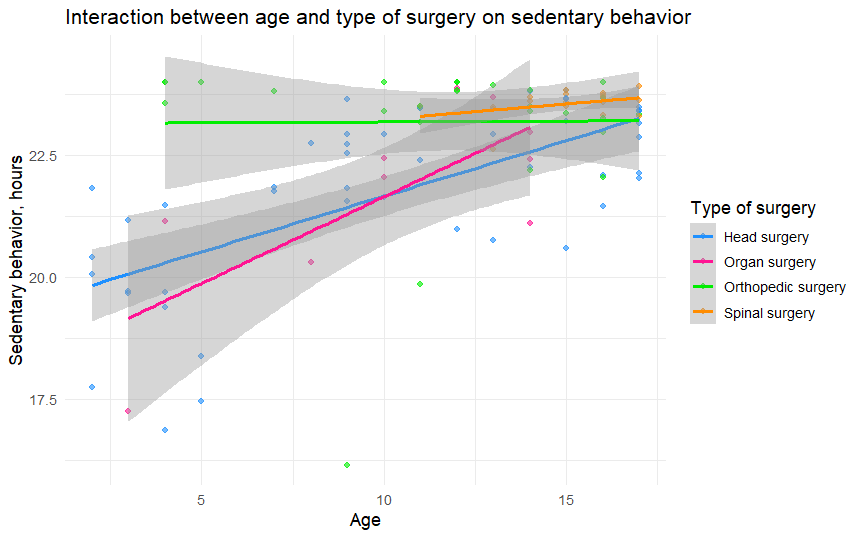


**Fig. B:** Interaction between age and type of surgery with confidence intervals. Interaction terms are reported in the main article.

| **Analysis of sedentary behaviour as a predictor of dichotomous outcome**  (Multiple logistic regression) | | | | |
| --- | --- | --- | --- | --- |
|  | **Odds ratio** | **CI 95** | **p-value** | **Goodness of fit** |
| **PRN opioid** |  |  |  |  |
| Adjusted for type of surgery and age | 1.75 | [1.06;2.90] | 0.028 | 0.528 |
| **Laxative use** |  |  |  |  |
| Adjusted for type of surgery, PRN opioid consumption, and age | 1.11 | [0.69;2.06] | 0.695 | 0.996 |
| **Readmission** |  |  |  |  |
| Adjusted for type of surgery and age | 0.82 | [0.47;1.41] | 0.467 | 0.812 |
| **Analysis of sedentary behaviour as a predictor of continuous outcome**  (Linear regression) | | | | |
| **Length of stay** | **ϐ-coefficient** | **CI 95** | **p-value** | **R^2^** |
| Adjusted for type of surgery and age | 0.134 | [-0.174;0.442] | 0.391 | 0.170 |

**Table h:** The association between sedentary behaviour and clinical outcomes. Results from logistic regression models predicting PRN opioid use, laxative use, and readmission status, and a linear regression model predicting length of stay, all adjusted for sedentary behaviour, age, and type of surgery. For logistic models, odds ratios (OR) with 95% confidence intervals (CI) are presented. Model fit was assessed using the Hosmer-Lemeshow test (p>0.05 indicates good fit). For the linear model, coefficients with 95% CI and R² are reported. Sedentary behaviour explained 17% of the variance in length of stay.
